# *Hdac6* Knock-Out Increases Tubulin Acetylation but Does Not Modify Disease Progression in the R6/2 Mouse Model of Huntington's Disease

**DOI:** 10.1371/journal.pone.0020696

**Published:** 2011-06-03

**Authors:** Anna Bobrowska, Paolo Paganetti, Patrick Matthias, Gillian P. Bates

**Affiliations:** 1 Department of Medical and Molecular Genetics, King's College London, London, United Kingdom; 2 Novartis Institutes for BioMedical Research, Neuroscience Discovery, Basel, Switzerland; 3 Friedrich Miescher Institute for Biomedical Research, Novartis Research Foundation, Basel, Switzerland; Thomas Jefferson University, United States of America

## Abstract

Huntington's disease (HD) is a progressive neurodegenerative disorder for which there is no effective disease modifying treatment. Following-on from studies in HD animal models, histone deacetylase (HDAC) inhibition has emerged as an attractive therapeutic option. In parallel, several reports have demonstrated a role for histone deacetylase 6 (HDAC6) in the modulation of the toxicity caused by the accumulation of misfolded proteins, including that of expanded polyglutamine in an N-terminal huntingtin fragment. An important role for HDAC6 in kinesin-1 dependent transport of brain-derived neurotrophic factor (BDNF) from the cortex to the striatum has also been demonstrated. To elucidate the role that HDAC6 plays in HD progression, we evaluated the effects of the genetic depletion of HDAC6 in the R6/2 mouse model of HD. Loss of HDAC6 resulted in a marked increase in tubulin acetylation throughout the brain. Despite this, there was no effect on the onset and progression of a wide range of behavioural, physiological, molecular and pathological HD-related phenotypes. We observed no change in the aggregate load or in the levels of soluble mutant exon 1 transprotein. HDAC6 genetic depletion did not affect the efficiency of BDNF transport from the cortex to the striatum. Therefore, we conclude that HDAC6 inhibition does not modify disease progression in R6/2 mice and HDAC6 should not be prioritized as a therapeutic target for HD.

## Introduction

Huntington's disease (HD) is an autosomal dominant progressive neurodegenerative disorder with a mean age of onset of 40 years [1]. The most characteristic features of symptomatic HD patients are motor disorders, cognitive decline, psychiatric disturbances and weight loss. The disease progresses on average for 15–20 years and although the first symptomatic drug has recently been approved by the Federal Drug Administration, there is still no disease modifying treatment available [2]. The cause of HD is the expansion of a CAG trinucleotide repeat in the *HTT* gene, resulting in an expanded polyglutamine (polyQ) tract in the N-terminus of the huntingtin protein [3]. HD pathology is mostly observed in the brain with the striatum displaying pronounced atrophy, although other brain regions are also affected [4], [5]. A prominent feature of HD is the presence of mutant huntingtin containing cytoplasmic aggregates and nuclear inclusions that are ubiquitin positive [6]. At the molecular level, many changes have been observed that may contribute to HD pathology including abnormal levels of neurotransmitters and their receptors, mitochondrial dysfunction, metabolic disturbances, transcriptional dysregulation and disruption of microtubule based transport, among others [7].

Histone deacetylases (HDACs) have been proposed as possible therapeutic targets for HD [8]. There are altogether 18 mammalian HDAC's, divided into four classes depending on their homology to yeast enzymes. Class I and II enzymes have Zn^2+^ dependent catalytic domains and are homologous to yeast Rpd3 and Hda1, respectively. HDAC11 is also Zn^2+^ dependent but has been placed in a separate category (class IV) due to a lack of sufficient sequence similarity to any other group [9]. Sirtuins (class III) are homologous to yeast Sir2 and use a different mechanism requiring NAD^+^ as a co-factor [10]. Studies on HDAC inhibition have shown promising results in fly, worm and mouse models of HD [11], [12], [13], [14].

HDAC6 is a target of some of the broad range HDAC inhibitors including suberoyl anilide hydroxamic acid (SAHA) and trichostatin A (TSA) [15]. It is a particularly interesting protein, in that it is the only known HDAC with two catalytically active deacetylase domains and a ubiquitin interacting domain [16], [17]. Moreover, its main activity appears to be in the cytoplasm, where it has been shown to deacetylate α-tubulin, HSP90 and cortactin, among others [18], [19], [20], [21]. HDAC6 is also of particular interest in HD. In cell models, HDAC6 has been shown to act against protein misfolding toxicity by taking part in the formation of a juxtanuclear structure termed the aggresome, a microtubule dependent inclusion body to which dispersed aggregates are targeted and transported by the dynein motor [22]. HDAC6 is required for the targeting of ubiquitinated aggregates to the aggresome, thought to serve as an adaptor protein by binding both poly-ubiquitin chains and the dynein motor. Interestingly, deacetylase activity and intact microtubules are essential to this process implying that aggresome formation depends on tubulin acetylation status [23]. Similarly, it has been shown that HDAC6 is critical for the formation of stress granules [24]. In addition, it has recently been shown that in the case of proteasome overload, aggresome formation might provide the means by which the cell removes accumulated misfolded proteins by autophagy and that HDAC6 is essential for this pathway [25]. In a *Drosophila melanogaster* model of spinal and bulbar muscular atrophy (SBMA) HDAC6 expression rescued neurodegeneration in an autophagy-dependent manner [26]. In fact, HDAC6 has also recently been shown to be involved in ubiquitin selective quality control autophagy by regulating the lysosome to autophagosome fusion [27]. On the other hand, a study in an HD cell model has indicated that increasing tubulin acetylation by HDAC6 inhibition could rescue the brain-derived neurotrophic factor (BDNF) transport deficit from the cortex to the striatum [28]. Taken together, these findings strongly suggest that HDAC6 inhibition should modify HD progression.

Based on the previous studies in cell culture models of HD, we might expect that loss of HDAC6 would increase aggregate load and exacerbate the phenotype in a mouse model of HD. On the other hand, an increase in cortico-striatal BDNF transport might be expected to improve some HD-related phenotypes. To determine whether HDAC6 regulates aggregate formation and turnover and/or plays a role in BDNF transport in a mouse model of HD, we crossed the R6/2 transgene onto an *Hdac6* knock-out background. As *Hdac6* is located on the X-chromosome, hemizygous male mice are nullizygous for *Hdac6* [29]. The R6/2 mouse has an early onset and rapid phenotype progression [30] and at late stage disease expresses HD-related phenotypes that are extremely similar to those that develop in the *Hdh*Q150 knock-in model of HD [31], [32], [33], [34], [35]. We showed that in the absence of HDAC6, levels of tubulin acetylation were increased throughout the brain. However, this had no effect on R6/2 readouts in a battery of physiological and behavioural assessments. We also found no difference in levels of soluble or aggregated huntingtin trans-protein upon ablation of HDAC6. Our data thus suggest that HDAC6 is not important for aggregate clearance in a mouse model of HD. Finally, we established that increased tubulin acetylation did not affect levels of BDNF in the striatum. We conclude that inhibition of HDAC6 should not be pursued as a high priority therapeutic target for HD.

## Results

**Figure 1:**
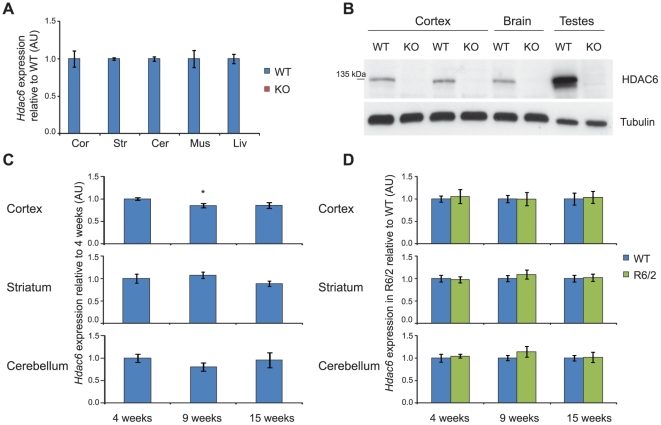
HDAC6 expression in *Hdac6*KO, WT and R6/2 mice. (**A**) Taqman qPCR assay showing absence of *Hdac6* mRNA expression in *Hdac6*KO mice as compared to WT; n = 3/genotype. Data normalised to *Atp5b* and expressed as fold change of WT for each tissue. (**B**) Western blot showing absence of HDAC6 protein expression in *Hdac6*KO mice as compared to WT. (**C**) Taqman qPCR assay showing that *Hdac6* mRNA expression does not change with age in WT mice in cortex, striatum and cerebellum; n = 8/genotype, data normalised to *Atp5b and Rpl13a* (cortex), *Gapdh* (striatum) and 18S RNA (cerebellum) and expressed as fold change of 4 weeks. (**D**) Taqman qPCR assay showing no difference in *Hdac6* mRNA expression between WT and R6/2 mice in cortex, striatum and cerebellum at 4, 9 and 15 weeks; n≥6/genotype, data normalised to WT for each time point and brain region. Error bars represent SEM. * p<0.05 as compared to 4 weeks. KO – *Hdac6* knock out, WT – wild type, Cor - cortex, Str - striatum, Cer - cerebellum, Mus - muscle, Liv - liver.

*Hdac6* knock-out mice (*Hdac6*KO) are viable and fertile, and do not show any overt phenotypes [36]. Before analysing the effect of the genetic depletion of HDAC6 on phenotype progression in the R6/2 mice, we wanted to verify that these mice do not express HDAC6 at the mRNA or protein level. cDNA was prepared from cortex, striatum, cerebellum, muscle and liver from 4 week old wild type (WT) and *Hdac*6KO mice and analysed by real-time quantitative RT-PCR (qPCR). *Hdac6* was not expressed in tissue from *Hdac6*KO mice (Fig. 1A). HDAC6 protein could not be detected in brain or testes by western blot analysis (Fig. 1B). Consistent with previous reports, HDAC6 levels were higher in testes than in brain [36]. R6/2 mice in our colony reach end-stage disease at 15 weeks of age. In order to determine whether *Hdac6* is stably expressed during this disease time-frame, we compared the levels of *Hdac6* mRNA in WT mice at 4, 9 and 15 weeks of age. We established that *Hdac6* is expressed at relatively stable levels in WT mice from 4 to 15 weeks of age, with only a 15% reduction being detected in the cortex at 9 weeks as compared to 4 weeks of age (Fig. 1C). Transcriptional dysregulation is a hallmark of the molecular pathogenesis of HD and is recapitulated by the R6/2 mouse model [37]. Thus, to ensure that *Hdac6* is not dysregulated in R6/2 mice, we measured *Hdac6* expression levels in R6/2 mice at 4, 9 and 15 weeks of age in cortex, striatum and cerebellum and compared them to those observed in WT mice. *Hdac6* levels were unchanged between WT and R6/2 mice (Fig. 1D) indicating that there was no effect of disease progression on the expression of *Hdac6*.

### Tubulin is hyperacetylated upon genetic depletion of HDAC6 but is not altered by the presence of the R6/2 transgene

**Figure 2:**
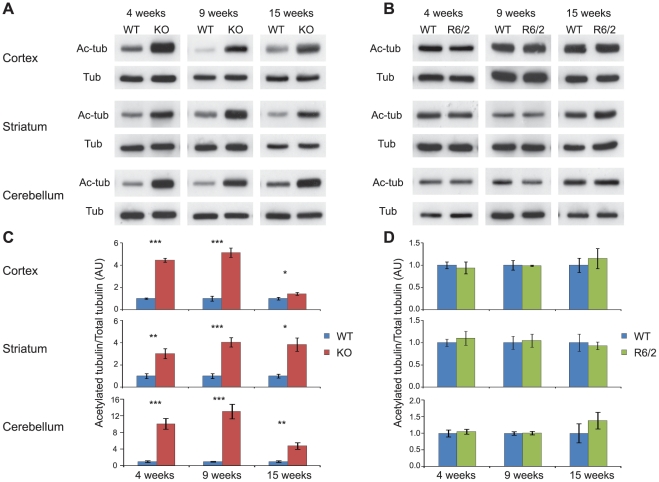
Tubulin acetylation in the brains of *Hdac6*KO and R6/2 mice compared to WT. (**A–B**) Representative western blots showing acetylated tubulin (Ac-tub) between *Hdac6*KO and WT mice (A) and R6/2 and WT mice (B) at 4, 9 and 15 weeks in cortex, striatum and cerebellum with tubulin (Tub) as a loading control. (**C–D**) Densitometric quantification of western blots represented in (A–B). Acetylated tubulin was normalised to α-tubulin and the relative signal for *Hdac6*KO (C) or R6/2 (D) expressed as fold change compared to WT. Error bars represent SEM. * p<0.05, ** p<0.01, *** p<0.001; n≥4/genotype. KO – *Hdac6* knock out, WT – wild type.

As HDAC6 is an α-tubulin deacetylase, depletion of HDAC6 should result in tubulin hyper-acetylation. It has been previously reported that the *Hdac6*KO line of mice used in this study, has elevated levels of acetylated tubulin in peripheral tissue but not in brain [36]. However, increased tubulin acetylation has been shown to occur in brain as a consequence of HDAC6 depletion in an alternative *Hdac6* knock-out mouse [38]. To resolve this discrepancy, we analysed tubulin acetylation in our *Hdac6*KO mice by western blotting, normalising levels of acetylated tubulin to those of total α–tubulin. We found that tubulin is hyperacetylated in the cortex, striatum and cerebellum of *Hdac6*KO mice at 4, 9 and 15 weeks of age as compared to WT littermates (Fig. 2A and C). The extent of hyperacetylation in *Hdac6*KO mice became less pronounced with age in the cortex and cerebellum, perhaps explaining the earlier results.

Acetylation of lysine 40 on α-tubulin has been demonstrated to increase the binding of certain molecular motor proteins to microtubules, thus modulating intracellular transport dynamics [39]. Impairment of intracellular transport has been suggested to contribute to the molecular pathology of HD [40]. Therefore, we investigated whether tubulin acetylation is altered by the presence of the R6/2 transgene. Our results indicate that there is no difference in tubulin acetylation between R6/2 and WT mice in cortex, striatum and cerebellum at 4, 9 or 15 weeks of age (Fig. 2B and D). This is consistent with the fact that there is no difference in the expression of *Hdac6* between WT and R6/2 mice in the same tissues throughout disease progression (Fig. 1D).

### Genetic Depletion of HDAC6 does not modify HD physiological and behavioural phenotypes in R6/2 mice

**Figure 3:**
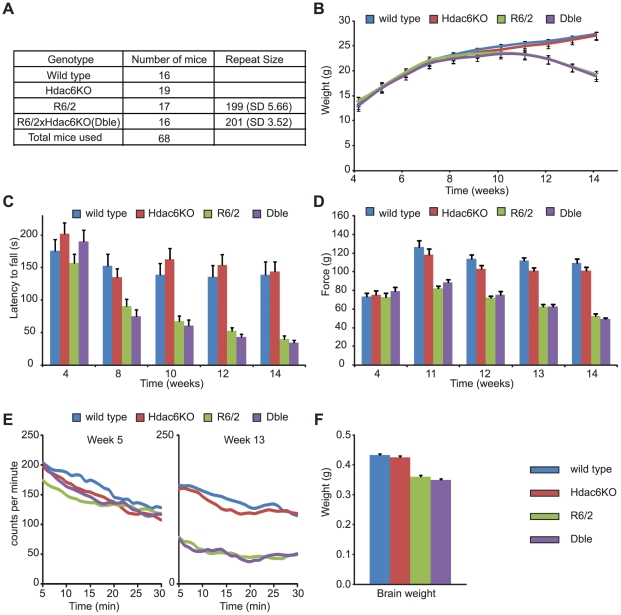
Behavioural and physiological phenotypes in the absence of HDAC6. (**A**) The number of male mice analysed per genotype with the mean CAG repeat size for the groups carrying the R6/2 transgene (SD-standard deviation). (**B**) Body weight (**C**) Time spent on RotaRod (**D**) Forelimb grip strength (**E**) Spontaneous motor activity recorded at the beginning (week 5) and end (week 13) of the study. Graphs were created by plotting 5 min moving averages (**F**) Brain weight measured at week 15. Error bars represent SEM.

Previous studies have indicated that both over-expression and depletion of HDAC6 could ameliorate HD phenotypes in cell culture [25], [28]. Here, we investigate whether HD-related phenotypes are modulated by the genetic depletion of HDAC6 in an HD mouse model. R6/2 males were bred to *Hdac6*KO heterozygous females and a longitudinal phenotyping study was performed to compare *Hdac6*KOxR6/2 double mutant (Dble) mice to WT, *Hdac6*KO and R6/2 littermates. As *Hdac6* is an X-linked gene [29], males carrying the mutant allele are effectively nullizygous for *Hdac6* (Fig. 1A–B). We obtained at least 16 males for each genotype and the R6/2 and Dble groups were well matched for their CAG repeat size (p = 0.338) (Fig. 3A).

Mice were weighed weekly to the nearest 0.1 g from 4 weeks of age onwards (Fig. 3B). R6/2 mice weighed significantly less than WT mice overall (F_(1,64)_ = 14.28, p<0.001) and gained weight at a significantly slower rate (F_(2,640)_ = 55.99, p<0.001). HDAC6 depletion by itself had no impact on the weight (F_(1,64)_ = 0.567, p = 0.454) and weight gain (F_(2,640)_ = 0.247, p = 0.741) when compared to WT mice. Absence of HDAC6 did not affect the loss of weight in R6/2 (F_(1,64)_ = 0.007, p = 0.931) or the decreased rate in weight gain (F_(2,640)_ = 0.512, p = 0.567). These data indicate that depletion of HDAC6 does not affect weight loss in the R6/2 mice (Fig. 3B).

RotaRod measures motor coordination and balance, both of which become significantly impaired in R6/2 mice. Here the animals were subjected to RotaRod analysis at 4, 8, 10, 12 and 14 weeks of age (Fig. 3C). As expected, R6/2 mice performed worse than WT mice (F_(1,71)_ = 39.74, p<0.001) and that performance deteriorated over the course of the study (F_(3,1420)_ = 18.23, p<0.001). The performance of *Hdac6*KO mice was similar to WT (F_(1,71)_ = 0.32, p = 0.573) but the variability in their performance over time was significantly different to that of WT mice (F_(3,1420)_ = 3.53, p = 0.020). Nevertheless, the *Hdac6* knock-out genotype did not modify the performance of R6/2 mice overall (F_(1,71)_ = 0.40, p = 0.529) or with age (F_(3,1420)_ = 1.09, p = 0.352).

Forelimb grip strength was measured at 4 weeks of age and weekly from 11 to 14 weeks of age (Fig. 3D). As expected, the overall grip strength of R6/2 mice was worse than that of WT (F_(1,67)_ = 142.41, p<0.001) and deteriorated over time (F_(3,536)_ = 50.69, p<0.001). The grip strength of *Hdac6*KO mice was no different from WT mice either overall (F_(1,67)_ = 0.621, p = 0.434) or with time (F_(3,536)_ = 1.97, p = 0.121). Nullizygosity for *Hdac6* did not have an effect on the overall grip strength of R6/2 mice (F_(1,67)_ = 3.17, p = 0.081) nor did it influence the deterioration in grip strength over the course of the experiment (F_(3,536)_ = 0.856, p = 0.463). Therefore, genetic depletion of HDAC6 does not modify loss of forelimb muscle strength in R6/2 mice.

Spontaneous motor activity was recorded for each mouse for 30 min in an infrared activity monitoring cage bi-weekly from 5 weeks of age onwards (Fig. 3E and Fig. S1). Data were analysed by repeated measures general linear model ANOVA and *p*-values are presented in Table S1. At 5 weeks of age, levels of activity between mice of all genotypes were comparable (Fig. 3E and Table S1: R6/2 Genotype and *Hdac6*KO Genotype), but a significant difference in the pattern of activity over time was already evident for activity (p = 0.011) and rearing (p = 0.016) for mice carrying the R6/2 transgene. At 7 weeks of age the R6/2 genotype significantly affected the activity and mobility (p<0.001) of the mice. A clear hypoactivity was evident in both the R6/2 and Dble mice at 9, 11 and 13 weeks of age (Fig. 3E and Table S1, R6/2 Genotype). *Hdac6*KO mice were comparable to WT and the Dble mutant mice to R6/2 at almost all time points indicating that depletion of HDAC6 had no influence on the R6/2 hypoactivity phenotype.

Mice were sacrificed at 15 weeks of age and brains weighed to the nearest 0.001 g (Fig. 3F). As expected, R6/2 mice had a significantly lower brain weight than WT mice (p<0.001). The weight of *Hdac6*KO brains was similar to WT (p<0.057) and that of the Dble mutant mice was similar to R6/2 (p = 0.784). Thus, absence of HDAC6 does not modify the loss in brain weight exhibited by the R6/2 mice at end-stage disease.

### Genetic Depletion of HDAC6 does not modify huntingtin aggregation

Aggregation of mutant huntingtin is a prominent feature of HD neuropathology and occurs in all of the HD rodent models that have thus far been developed. Aggregates in the R6/2 mouse appear prior to the onset of behavioural symptoms and continue to accumulate throughout the course of the disease. At the same time, a steady decrease in the levels of soluble transprotein can be observed. In cells, HDAC6 acts as a linker between ubiquitinated protein and/or aggregates and the dynein/dynactin motor complex enabling their transport to the aggresome for autophagic degradation [23]. If this phenomenon is relevant to the mammalian brain, an absence of HDAC6 should result in an increase in aggregate load [25].

**Figure 4:**
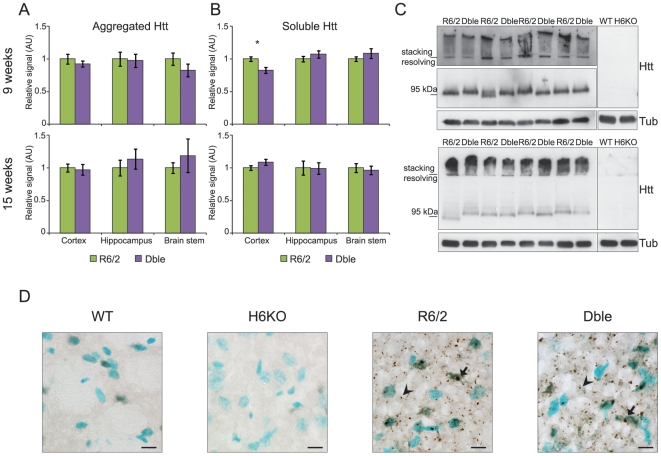
No effect of *Hdac*6 knock-out on aggregate load or soluble transprotein levels. (**A**) Aggregate load was measured by Seprion ligand ELISA (MW8 antibody) and (**B**) soluble transprotein levels were measured by TR-FRET (2B7-MW1 antibodies) in the cortex, hippocampus and brain stem of R6/2 and Dble mice at 9 and 15 weeks. The average of the WT and *Hdac6*KO background signal was subtracted from both R6/2 and Dble signals. The Dble signal is expressed as fold change of the R6/2 signal. * p<0.05; n≥8/genotype. (**C**) Representative western blots with S830 anti-huntingtin antibody showing aggregated and soluble transprotein (Htt) in the cortex at 9 (upper panel) and 15 (lower panel) weeks. α-tubulin (Tub) was used as a loading control. Specificity of S830 staining is confirmed by lack of signal in WT and *Hdac6*KO cortices. (**D**) Representative sagittal sections from 9 week old mice showing the striatum immunostained with S830 antibody. Nuclei are counterstained with methyl green. Scale bar = 10 µm. Arrow = nuclear inclusion, arrowhead = cytoplasmic aggregate. Error bars represent SEM.

We measured the amount of SDS insoluble aggregates by the Seprion ligand ELISA [34] and found no difference in aggregate load between R6/2 and Dble mice at 4, 9 and 15 weeks of age in cortex, hippocampus and brain stem (Fig. 4A and Fig. S2A), or at 9 and 15 weeks in quadriceps muscle (Fig. S2E). The corresponding levels of soluble transprotein were measured in these tissues by means of TR-FRET (Fig. 4B and Fig. S2D–E), similarly, no difference between R6/2 and Dble mice was detected. These results were confirmed by a TR-FRET aggregation assay (Fig. S2D) and by western blotting of SDS-PAGE resolved lysates with an antibody that detects soluble and aggregated huntingtin (S830) (Fig. 4C and Fig. S2B).

Our data show that HDAC6 ablation does not influence aggregate load or levels of soluble mutant huntingtin in the cortex, hippocampus or brain stem at 4, 9 or 15 weeks of age (Fig. 4, Fig. S2A–C) or in quadriceps muscle at 9 and 15 weeks of age (Fig. S2E). In support of this, we did not detect any obvious differences in the level or distribution of aggregates between R6/2 and Dble striata at 9 weeks of age by immunohistochemistry (Fig. 4D). Although a significant decrease in soluble cortical transprotein levels was detected by TR-FRET in the Dble as compared to the R6/2 mice at 9 weeks of age, further analysis by western blot has shown that this difference, if real, is not large (Fig. 4B–C). At the same age, there was no difference in the cortical aggregate load measured by either Seprion ELISA or TR-FRET (Fig. 4A and Fig. S2D).

### Genetic Depletion of HDAC6 results in tubulin hyperacetylation in the brains of R6/2 mice

**Figure 5:**
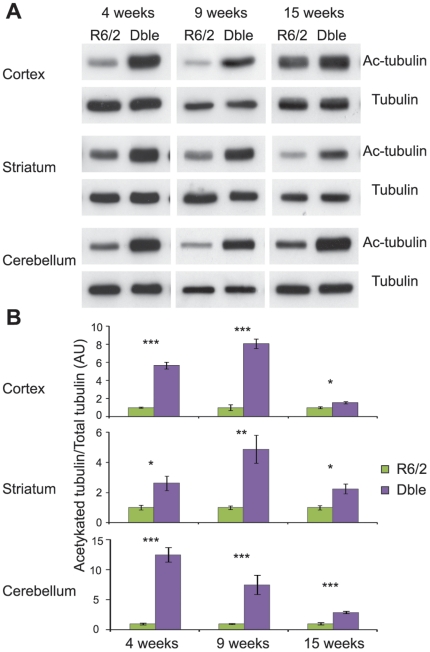
Tubulin acetylation in the brains of Dble as compared to R6/2 mice. (**A**) Representative western blots showing acetylated tubulin in Dble and R6/2 mice at 4, 9 and 15 weeks in cortex, striatum and cerebellum with α-tubulin as a loading control. (**B**) Densitometric quantification of western blots presented in (A). Acetylated tubulin was normalised to α-tubulin and the relative signal for Dble mice expressed as fold change to R6/2. Error bars represent SEM. * p<0.05, ** p<0.01, ***p<0.001; n≥4/genotype.

We have found that the absence of HDAC6 has no effect on R6/2 behavioural phenotypes or on the accumulation of aggregated transprotein in these animals. It is possible that the presence of the R6/2 transgene prevented the hyperacetylation of tubulin from occurring in response to HDAC6 depletion. Therefore, we compared the levels of acetylated tubulin between R6/2 and Dble mice at 4, 9 and 15 weeks of age in the cortex, striatum and cerebellum. We found that HDAC6 depletion resulted in a pronounced increase in tubulin acetylation in R6/2 mice in all brain regions and at all time-points studied (Fig. 5). The increase in tubulin acetylation was comparable between *Hdac6*KO and Dble mutant mice (Fig. S3). We conclude that the substantial increase in tubulin acetylation levels throughout the brain has no consequence on HD-related phenotypes in the R6/2 mouse.

### Genetic Depletion of HDAC6 does not modify BDNF content in the striatum

**Figure 6:**
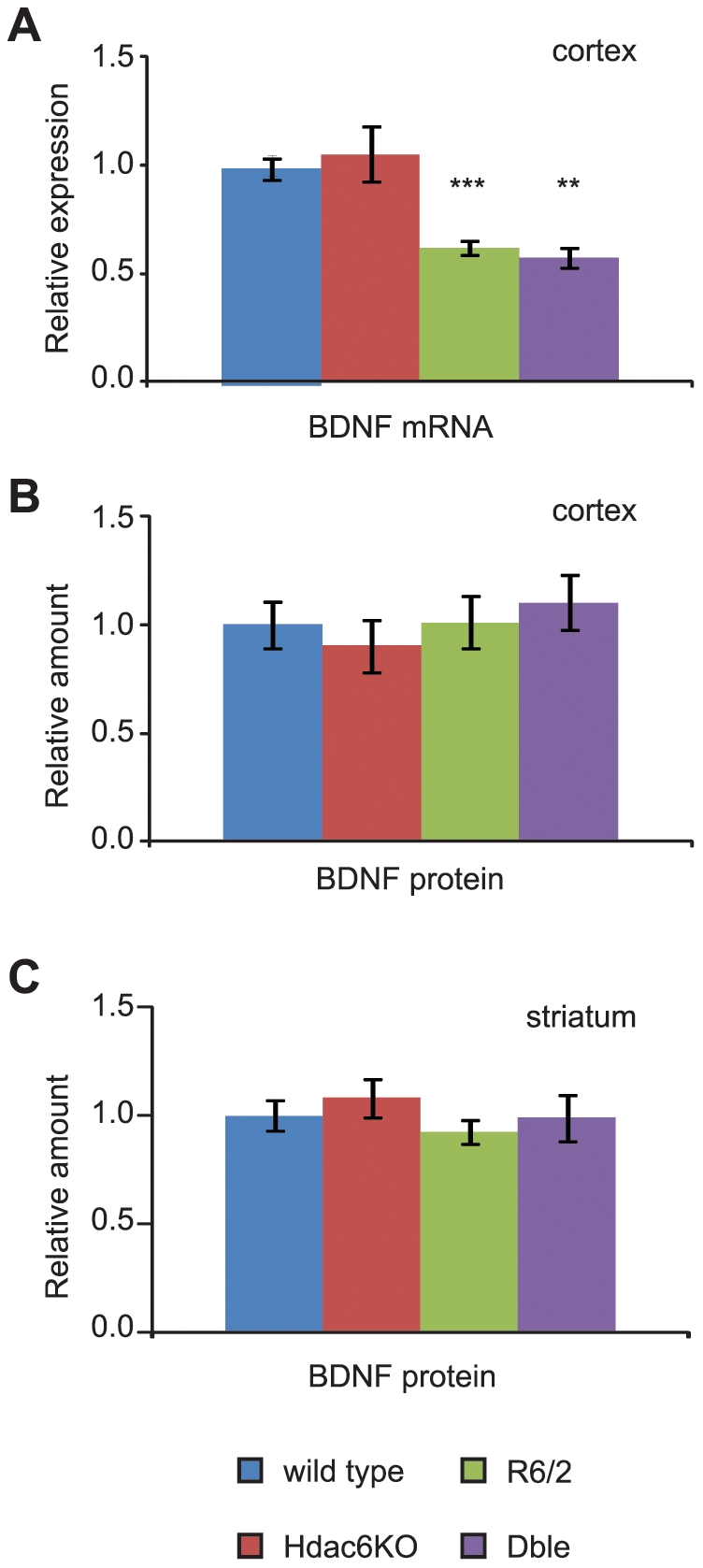
Influence of *Hdac*6 knock-out on *Bdnf* mRNA and protein levels. (**A**) Taqman qPCR assay showing a significant down-regulation of *Bdnf* coding region mRNA expression in cortex of mice carrying the R6/2 transgene. Data normalised to WT. ** p<0.01 (to *Hdac6KO*), ***p<0.001 (to WT); n≥4/genotype. (**B–C**) BDNF protein content in the cortex (B) and striatum (C) measured by ELISA. Data normalised to WT. n≥3/genotype in (B) and n≥4/genotype in (C). Error bars represent SEM.

Cell culture studies have shown that an increase in tubulin acetylation increases the transport of BDNF from the cortex to the striatum in the context of both WT and HD [28]. In accordance with previous data [41], we found that *Bdnf* mRNA expression is lower in the cortex of R6/2 mice at 9 weeks of age and that this phenotype is not altered by HDAC6 depletion (Fig. 6A). However, at the same age, levels of BDNF protein in the cortex as measured by ELISA were comparable between mice of all four genotypes (Fig. 6B). Equally, BDNF protein levels in the striatum of *Hdac6*KO, R6/2 and Dble mice were not different from those of WT mice (Fig. 6C). Therefore, the decrease in *Bdnf* mRNA did not translate to a reduction in BDNF protein. As *Bdnf* is expressed in the striatum at a very low level (Fig. S4A) and it has been published that most striatal BDNF protein originates in the cortex [42], we conclude that there is no effect of HDAC6 ablation on the transport efficiency of BDNF from the cortex to the striatum in either WT or R6/2 mice.

## Discussion

Previous studies in cell culture models of HD have shown that modulation of HDAC6 levels alters HD-related phenotypes [25], [28]. To extend this analysis to a mouse model, we investigated the effects of HDAC6 genetic depletion in the R6/2 mouse. We showed that *Hdac6* knock-out mice (*Hdac6*KO) do not express HDAC6 at the mRNA or protein levels and have hyperacetylated tubulin in the cortex, striatum and cerebellum from 4 to 15 weeks of age. We show that in the presence of the polyQ expanded R6/2 transprotein, levels of acetylated tubulin do not differ from that in wild type animals and similarly, the HD mutation does not interfere with the effect that HDAC6 ablation has on tubulin hyperacetylation. We found that depletion of HDAC6 had no effect on the aggregate load or BDNF levels in the R6/2 brain and did not modify physiological or behavioural HD-related phenotypes in R6/2 mice.

In this study, we have used mice in which the endogenous *Hdac6* gene has been disrupted [36]. One possible concern with using mice that do not express HDAC6 throughout development is that the expression of other genes will adjust to compensate for the deficit. The HDAC6 mechanisms of action under investigation here are based on HDAC6-tubulin interactions. Our demonstration that tubulin acetylation is increased in the brain from 4 to 15 weeks of age indicates that compensation has not occurred for the HDAC6 target in which we are primarily interested and that the constitutive *Hdac6* knock-out mice used here are a suitable model. There are numerous mouse models of HD, which include mice transgenic for an N-terminal fragment of *HTT*, YAC and BAC mice that are transgenic for full-length *HTT* gene and knock-in mice in which an expanded CAG repeat has been introduced into the mouse *Hdh* gene [43]. The HD cell culture model used by Iwata and colleagues [25] to show that HDAC6 is required for aggresome formation expressed an exon 1 huntingtin transprotein. The R6/2 mice express an exon 1 N-terminal fragment of mutant huntingtin [30] and therefore, they represent an appropriate model with which to first follow-up on their findings. Also, recent analysis has shown that the R6/2 mice develop HD-related phenotypes that are highly comparable to a genetically accurate knock-in mouse model of HD [31], [33], [34], [35] and that, in these knock-in mice, mutant huntingtin is processed to generate N-terminal fragments [32]. Despite this, analysing the effects of the modulation of *Hdac*6 levels on HD phenotypes in these more genetically precise models might be considered important. However, before embarking on such a project, it would be wise to determine whether a reduction in HDAC6 levels has an effect in cell culture models expressing a mutant version of full-length huntingtin.

HDAC6 has been extensively characterised as being required for the formation and subsequent autophagic degradation of the aggresome [23], [25], [27]. In an HD cell culture model, HDAC6 was required for aggresome formation and an increase in aggregate load was observed upon HDAC6 knock-down [25]. Proteasome inhibition resulted in an increase in ubiquitin positive aggregates in *Hdac6*KO mouse embryonic fibroblasts as compared to wild type cells indicating that HDAC6 is important for autophagy [44]. HDAC6 knock-down in a *Drosophila melanogaster* model of SBMA exacerbated retinal degeneration and overexpression of HDAC6 decreased aggregate load and ameliorated retinal degeneration, effects that required HDAC6 catalytic activity [26]. In keeping with a role for HDAC6 in protein homeostasis, ubiquitin positive aggregates have been reported to accumulate in the brains of another strain of *Hdac6*KO mice by 6 months of age [27]. Given that an increase in aggregation was not detected in R6/2 mice that lack HDAC6, we conclude that either quality-control autophagy is not important for the clearance of polyQ aggregates or that HDAC6 is not essential for this process in the R6/2 mouse brain. It is possible that the mechanism of aggregate handling could differ between lower organisms/cell culture models and HD mice.

In cultured cells, an increase in tubulin acetylation has been shown to enhance kinesin-1 binding and microtubule-based transport [39]. BDNF is a kinesin-1 cargoe [28], which is actively transported from the cortex to the striatum [42]. A recent study in cells has shown that by increasing tubulin acetylation one can increase BDNF cortico-striatal transport and that this is an HDAC6 dependent process [28]. In HD there is a well documented decrease in cortical *BDNF* mRNA expression [41] and we have confirmed that R6/2 mice at 9 weeks of age recapitulate this phenotype, regardless of presence or absence of HDAC6. However, this did not translate into a reduction in BDNF in either the cortex or the striatum in accordance with some, but not all previously published data [45], [46], [47], suggesting that R6/2 mice at 9 weeks do not display a deficit in BDNF transport. If HDAC6 depletion increases BDNF transport independent of the presence of the huntingtin mutation, as has been reported [28], we would expect to see an increase in striatal BDNF protein levels in both *Hdac6*KO and double mutant mice. We did not observe any change in BDNF levels. Strategies that increase BDNF levels have been shown to be beneficial in R6/2 mice and other N-terminal fragment models [45], [48], [49], [50]. If small changes had occurred, that were beyond the sensitivity of our detection method, they were not sufficient to improve the phenotype of R6/2 mice.

There has been an increased focus on HDAC6 in neurodegenerative disease. In addition to the polyglutamine diseases, over-expression of HDAC6 was protective in *Drosophila melanogaster* models of Parkinson's disease [51] and Alzheimer's disease [52]. HDAC6 has also been found to localise to Lewy bodies in Parkinson's disease patient brains [23] and there is also evidence supporting a role for HDAC6 in Alzheimer's disease via its association with tau [53]. Our finding that the knock-out of HDAC6 does not affect the phenotype, aggregate load or BDNF transport in R6/2 mice was very surprising. This study underlines the importance of validating pathogenic mechanisms and therapeutic targets in mammalian models. At the same time, our findings indicate that the protective effect of broad range HDAC inhibitors that has been observed in invertebrate and mouse HD models [8] is not predominantly mediated via inhibition of HDAC6. The current study is part of a wider project to investigate the effects of the genetic depletion (knock-out or knock-down) of specific HDACs on HD-related phenotypes in the R6/2 mouse. Genetic reduction of *Hdacs3*, *5*, *7* and *9* have not resulted in a phenotypic improvement ([54] and unpublished data) whereas knock-down of *Hdac4* has shown beneficial effects (unpublished data). Based on our data, we can conclude that HDAC6 inhibition would not be a valid therapeutic strategy for HD.

## Materials and Methods

### Ethics statement

All experimental procedures performed on mice were approved by the King's College London Ethical Review Process Committee and carried out under the UK Home Office License 70/6545.

### Mouse strains and husbandry

Hemizygous R6/2 mice were maintained by backcrossing R6/2 males to CBAxC57BL/6 F1 (CBF) females (B6CBAF1/OlaHsd, Harlan Olac, UK) [30]. *Hdac6* knock-out (*Hdac6*KO) mice [36] on C57BL/6 background were backcrossed once to CBF. For the R6/2x*Hdac6*KO genetic cross, R6/2 males were bred to *Hdac6* heterozygous females. At 4 weeks of age, mice were weaned into cages of 5, each containing at least one representative of each genotype. Animals were housed under 12 h light/12 h dark cycle, with unlimited access to water and chow (Special Diet Services, Witham, UK). Cages were environmentally enriched as described [55]. Mice from the R6/2x*Hdac6*KO cross were given mash food consisting of powdered chow mixed with water during 4–6 and 12–15 weeks of age and sacrificed at 15 weeks.

### Genotyping

Mice were genotyped by PCR of tail-tip DNA. R6/2 mice were genotyped and their repeat sizes determined as described [34]. For the *Hdac6* genotyping, the primers were forward: 5′-GTACAATGTGGCTCACAGAA, reverse wt: 5′-CAGGCACAGGAATATGAGTT and reverse KO: 5′-CAACTCTGCCTCTCCTGG each used 1 µL at 10 µM in one multiplex 10 µL reaction, containing also 1 µL of 100 ng/µL DNA, 0.8 µL 25 mM MgCl_2_, 1 µL 2 mM dNTP, 1 µL Sigma 10×PCR buffer and 0.1 µL Sigma Taq Polymerase (D4545, Sigma). Cycling conditions were as follows: 94°C for 5 min, (94°C for 30 s, 64°C for 30 s and 72°C for 1 min)×40 followed by 10 min at 72°C.

### Phenotypic assessment

The phenotypes of the mice from the R6/2x*Hdac6*KO cross were assessed blind to genotype. Mice were weighed weekly to the nearest 0.1 g. RotaRod performance was measured at 4 weeks of age for 4 consecutive days, 3 runs a day and after that at 8, 10, 12 and 14 weeks of age for 3 consecutive days, 3 runs a day, using an accelerating (4–44 rpm in 5 min) Ugo Basile 7650 Rotarod, (Linton Instrumentation, UK) modified as described [55]. Exploratory, spontaneous motor activity was recorded at 5, 7, 9, 11 and 13 weeks of age by placing mice in AM1053 activity cages for 30 min during the day, as described previously [56]. Activity was the total number of lower level beam breaks. Mobility was the number of at least two consecutive beam breaks occurring in the lower level. Rearing was the number of rearing beam breaks and centre rearing was the number of rearing beam breaks occurring away from the cage walls. Forelimb grip strength was assessed at 4 weeks of age and then weekly from 11 to 14 weeks, always prior to RotaRod measurements, with San Diego Instruments Grip Strength Meter (San Diego, CA, USA) as described previously [55]. Mice were sacrificed at 15 weeks of age and brains were weighed to the nearest 0.001 g.

### Antibodies

The HDAC6 antibody was a kind gift from Dr. Tso-Pang Yao [38]. Acetylated α-tubulin (6-11B-1, T7451) and α-tubulin (DM1A, T9026) antibodies were purchased from Sigma. S830 is a sheep polyclonal antibody raised against a GST tagged huntingtin exon 1 with 53 glutamines, characterized elsewhere [57] and was generated at Scottish Antibody Production Unit. MW8, MW1 [58] and 2B7 [59] were obtained from Novartis, Basel. Secondary peroxidase coupled antibodies were purchased from Dako (anti-goat, anti-mouse), Pierce (anti-rabbit) or KPL (anti-mouse for Seprion).

### Sample preparation

For tubulin acetylation analysis, tissues were homogenised using 1% Triton X-100 buffer with 50 mM Tris-HCl pH 7.5, 150 mM NaCl, 2 mM EDTA, 5 µM TSA (Trichostatin A, Sigma) and 10 mM nicotinamide (Sigma), supplemented with protease inhibitor cocktail (Roche). For HDAC6 immuno-detection, tissues were homogenised in RIPA buffer (1% NP-40, 0.5% Deoxycholate, 0.1% SDS, 50 mM Tris-HCl pH 8, 150 mM NaCl, 1 mM β-mercaptoethanol, 100 µM PMSF, 1 mM DTT) supplemented with protease inhibitor cocktail (Roche). Samples were sonicated on ice for 10 s at 80 Hz (Vibracell Sonicator). Lysates were cleared by centrifugation at 16 200 rcf for 15 min at 4°C. Protein concentration was measured with Pierce BCA assay kit (Thermo Scientific). Samples were diluted with 2× protein Laemmli buffer (1 M Tris-HCl pH 6.8, 2.3% SDS, 4.5% glycerol, 10% β-mercaptoethanol, 0.001 g/mL bromophenol blue) and denatured for 5 min at 95°C.

### Western blotting

Equal amounts of protein were loaded onto SDS polyacrylamide gel with a size reference (Broad Range Protein Marker, Cell Signalling or Spectra Broad Range Protein Ladder, Fermentas). Proteins were transferred onto Protran nitrocellulose membrane (Whatman) at 120 V for 90 min by submerged transfer apparatus (Bio-Rad) in transfer buffer (20% v/v methanol, 25 mM Tris, 192 mM glycine). Membranes were blocked in 5% non-fat dried milk in PBS for at least 1 hour. Primary antibodies were applied in 0.02% PBS-Tween 20 (PBST) for 20 min (1∶40000 acetylated α-tubulin; 1∶30000 α-tubulin) or 1 hour (1∶5000 S830) at room temperature or overnight (1∶250 HDAC6) at 4°C. Blots were washed thrice for 5 min in 0.2% PBST and incubated with appropriate HRP coupled secondary antibody (all 1∶5000 except anti-rabbit 1∶20000). For signal detection, GE Healthcare Enhanced Chemiluminescence detection system and Amersham Hyperfilms (both GE Healthcare) were used according to the manufacturer's instructions. Signals were quantified using a GS-800 densitometer (Bio-Rad).

### Aggregate detection with Seprion ligand ELISA

For aggregate detection 2.5% lysates (w/v) were prepared by homogenising tissue in RIPA. Aggregate capture and detection were performed in Seprion ligand coated plates (Microsens) as described [34].

### TR-FRET

Time resolved - Förster resonance energy transfer experiments (TR-FRET) were performed as described [60].

### BDNF ELISA

BDNF protein content was measured by the commercially available ELISA kit (Promega) according to manufacturer's instructions modified as described [61]. Lysates were prepared at 2.2% dilution (w/v) and were not acid treated.

### Taqman real time quantitative PCR

RNA extraction, cDNA synthesis, Taqman RT-qPCR and ΔCt analysis were performed as described previously [62]. Housekeeping genes (primer and probe mix purchased from Primer Design) were chosen appropriate to the brain region analysed. For time-course or across tissue analysis, several housekeeping genes were tested and the most stable ones chosen. Primers for *Hdac6* expression analysis were forward: 5′ - GGAGACAACCCAGTACATGAATGAA; reverse: 5′ - CGGAGGACAGAGCCTGTAG and the probe was 5′-FAM-TATCTGCATCCGAACTCATATTCCTGTGCCTG-TAMRA. Primers for *Bdnf* coding region were forward: 5′ - GGGTCACAGCGGCAGATAAA; reverse: 5′ - GCCTTTGGATACCGGGACTT; and the probe was: 5′ - FAM - TCTGGCGGGACGGTCACAGTCC - TAMRA.

### Immunohistochemistry

Whole brains were snap frozen in isopentane at −50°C. Immunohistochemistry was performed as described [31].

### Statistical Analysis

Data from the R6/2x*Hdac6*KO cross were analysed with SPSS using one way ANOVA or General Linear Model ANOVA with Greenhouse-Geisser correction for non-sphericity. Data from qPCR and tubulin acetylation assay were analysed with Microsoft Excel using Student's *t*-test (two tailed).

## Supporting Information

Figure S1 ***Hdac6*** **knock-out has no effect on spontaneous motor activity in WT or R6/2 mice.** Five minute moving averages for Activity, Mobility, Rearing and Centre rearing at 5, 7, 9, 11 and 13 weeks of age for WT, *Hdac6KO*, R6/2 and Dble mice. n≥16/genotype (as shown in Fig. 3A).
(TIF)

Figure S2 ***Hdac6*** **knock-out does not change aggregate load in the brain or muscle.** (**A**) Aggregate load was measured by Seprion ligand ELISA with the MW8 antibody in the cortex, hippocampus and brain stem of R6/2 and Dble mice at 4 weeks. n≥5/genotype. (**B**) Representative western blot with S830 anti-huntingtin antibody showing aggregated and soluble transprotein (Htt) in the cortex at 4 weeks. α-tubulin (Tub) was used as a loading control. Specificity of S830 staining is confirmed by lack of signal in WT and *Hdac6*KO cortices. (**C**) Densitometric quantification of soluble Htt from western blots shown in (A) and Fig. 4C. Htt signal was normalised to α-tubulin and the relative signal for Dble mice expressed as fold change to R6/2 for each time point. (**D**) Soluble (2B7-MW1 antibodies, upper panel) and aggregated (MW8-MW8 antibodies, lower panel) transprotein levels were measured by TR-FRET in the cortex at 9 and 15 weeks. Data normalised to R6/2 at 9 weeks. * p<0.05; n≥8/genotype. (**E**) Aggregate load was measured by Seprion ligand ELISA with the MW8 antibody and soluble transprotein levels were measured by TR-FRET (2B7-MW1 antibodies) in the quadriceps muscle at 9 and 15 weeks. Data normalised to R6/2 at each time point. n≥7/genotype. Error bars represent SEM.
(TIF)

Figure S3 **The increase in tubulin acetylation in brain is comparable between Hdac6KO and Dble mice.** (**A**) Representative western blots showing acetylated tubulin (Ac-tub) in Dble and *Hdac6*KO mice at 4, 9 and 15 weeks in cortex, striatum and cerebellum with α-tubulin (Tub) as a loading control. There was insufficient tissue to perform the analysis on striatum at 9 and 15 weeks. (**B**) Densitometric quantification of western blots presented in (A). Acetylated tubulin was normalised to α-tubulin and the relative signal for Dble mice expressed as fold change to *Hdac6*KO. Error bars represent SEM. KO - *Hdac6*KO, Dble - *Hdac6*KOxR6/2; n≥3/genotype.
(TIF)

Figure S4 **Similar levels of BDNF protein in striatum, cortex and hippocampus despite very low** ***Bdnf*** **mRNA striatal expression.** (**A**) *Bdnf* mRNA coding region expression in WT mice at 9 weeks between cortex, striatum and hippocampus. Data normalised to *Ubc* and expressed as fold change of cortex. n≥3/genotype. ** p<0.01 (to cortex) *** p<0.001 (to cortex and to hippocampus) (**B**) BDNF protein content measured by ELISA in cortex, striatum, hippocampus and liver in WT mice at 9 weeks of age. Data normalised to cortex. * p<0.05; n = 3/genotype. Error bars represent SEM.
(TIF)

Table S1 Statistical analysis of the influence of time (30 min duration) and genotype(s) on the spontaneous motor activity parameters: activity, mobility, rearing and centre rearing presented as *p* - values calculated via ANOVA General Linear Model with Greenhouse-Geisser correction.
(DOC)
