# Supplementary figures and images for: Hdac6 Knock-Out Increases Tubulin Acetylation but Does Not Modify Disease Progression in the R6/2 Mouse Model of Huntington's Disease
Source: PLoS One. 2011 Jun 3;6(6):e20696. doi: 10.1371/journal.pone.0020696 (PMC3108987; doi:10.1371/journal.pone.0020696)

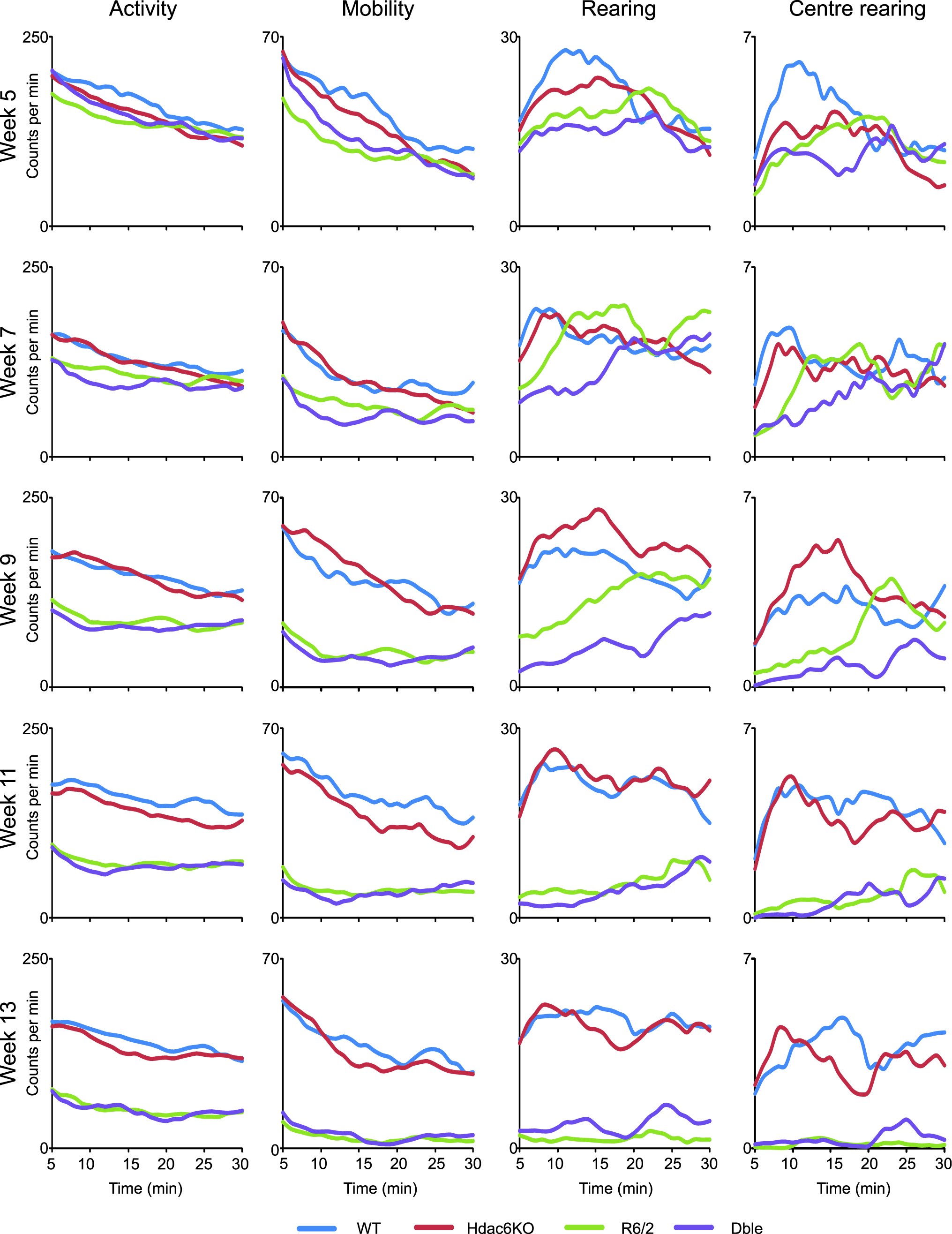

Supplement: Figure S1 — Hdac6 knock-out has no effect on spontaneous motor activity in WT or R6/2 mice. Five minute moving averages for Activity, Mobility, Rearing and Centre rearing at 5, 7, 9, 11 and 13 weeks of age for WT, Hdac6KO, R6/2 and Dble mice. n≥16/genotype (as shown in Fig. 3A). (TIF) [file pone.0020696.s001.tif]

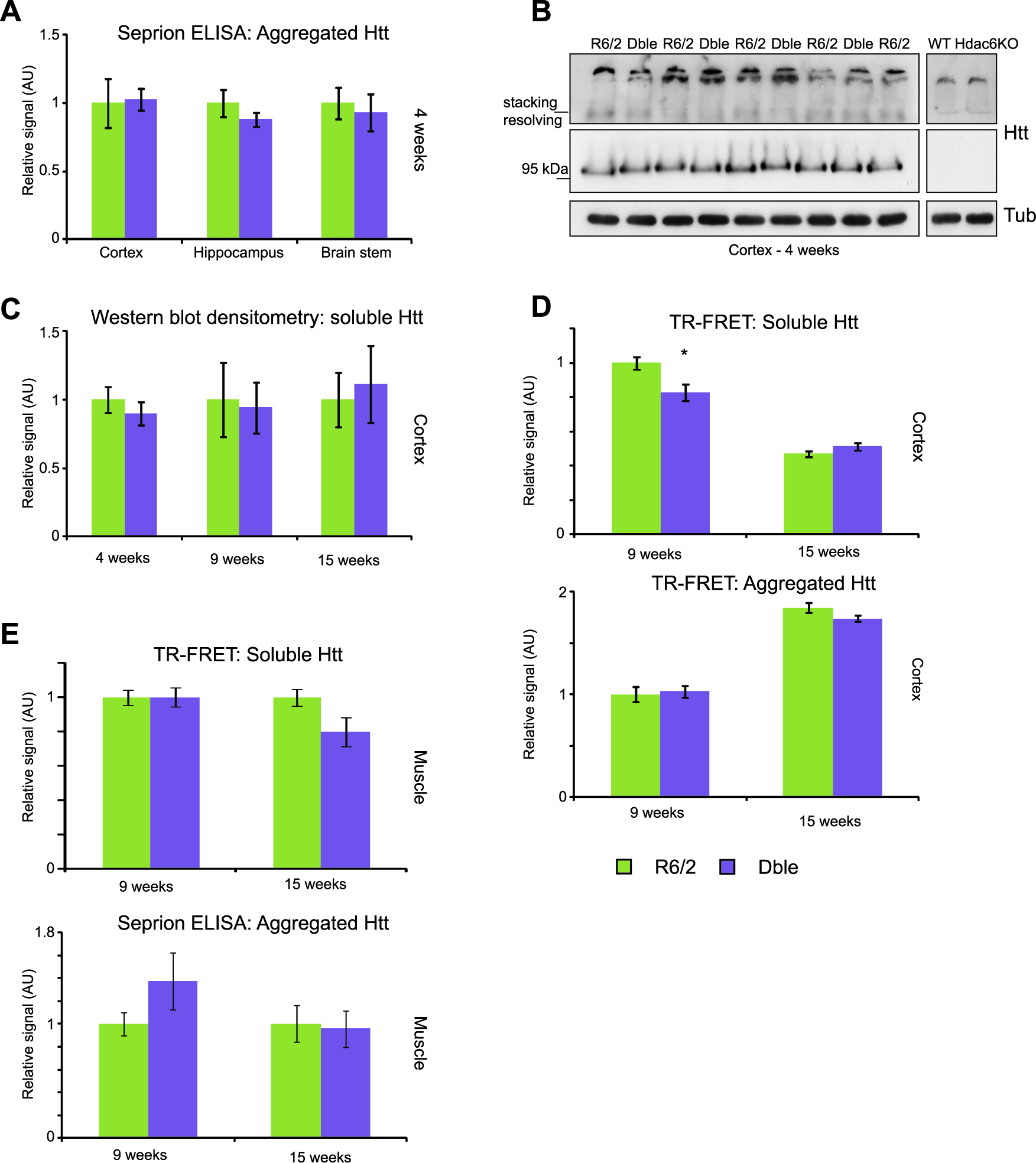

Supplement: Figure S2 — Hdac6 knock-out does not change aggregate load in the brain or muscle. (A) Aggregate load was measured by Seprion ligand ELISA with the MW8 antibody in the cortex, hippocampus and brain stem of R6/2 and Dble mice at 4 weeks. n≥5/genotype. (B) Representative western blot with S830 anti-huntingtin antibody showing aggregated and soluble transprotein (Htt) in the cortex at 4 weeks. α-tubulin (Tub) was used as a loading control. Specificity of S830 staining is confirmed by lack of signal in WT and Hdac6KO cortices. (C) Densitometric quantification of soluble Htt from western blots shown in (A) and Fig. 4C. Htt signal was normalised to α-tubulin and the relative signal for Dble mice expressed as fold change to R6/2 for each time point. (D) Soluble (2B7-MW1 antibodies, upper panel) and aggregated (MW8-MW8 antibodies, lower panel) transprotein levels were measured by TR-FRET in the cortex at 9 and 15 weeks. Data normalised to R6/2 at 9 weeks. * p<0.05; n≥8/genotype. (E) Aggregate load was measured by Seprion ligand ELISA with the MW8 antibody and soluble transprotein levels were measured by TR-FRET (2B7-MW1 antibodies) in the quadriceps muscle at 9 and 15 weeks. Data normalised to R6/2 at each time point. n≥7/genotype. Error bars represent SEM. (TIF) [file pone.0020696.s002.tif]

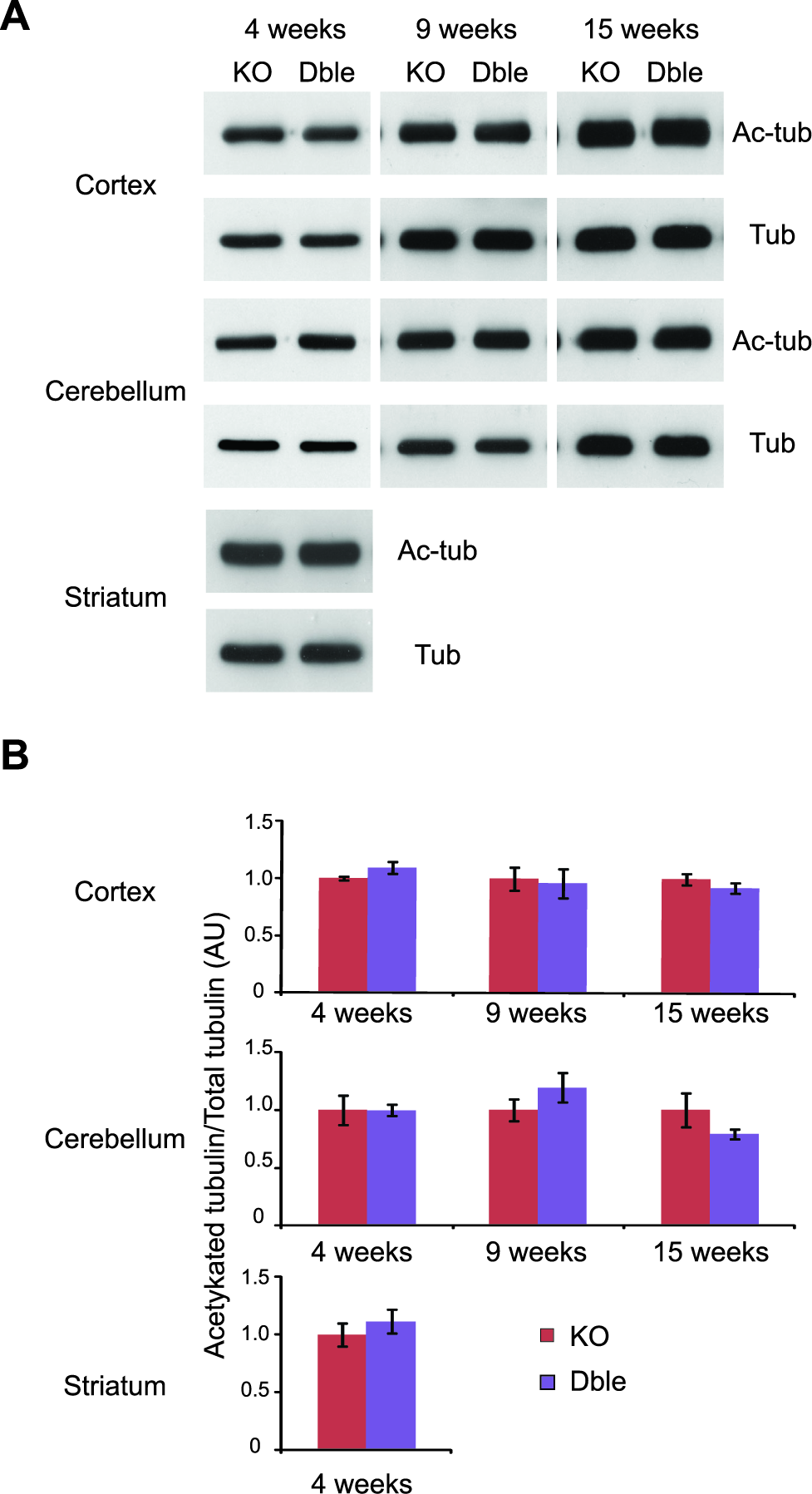

Supplement: Figure S3 — The increase in tubulin acetylation in brain is comparable between Hdac6KO and Dble mice. (A) Representative western blots showing acetylated tubulin (Ac-tub) in Dble and Hdac6KO mice at 4, 9 and 15 weeks in cortex, striatum and cerebellum with α-tubulin (Tub) as a loading control. There was insufficient tissue to perform the analysis on striatum at 9 and 15 weeks. (B) Densitometric quantification of western blots presented in (A). Acetylated tubulin was normalised to α-tubulin and the relative signal for Dble mice expressed as fold change to Hdac6KO. Error bars represent SEM. KO - Hdac6KO, Dble - Hdac6KOxR6/2; n≥3/genotype. (TIF) [file pone.0020696.s003.tif]

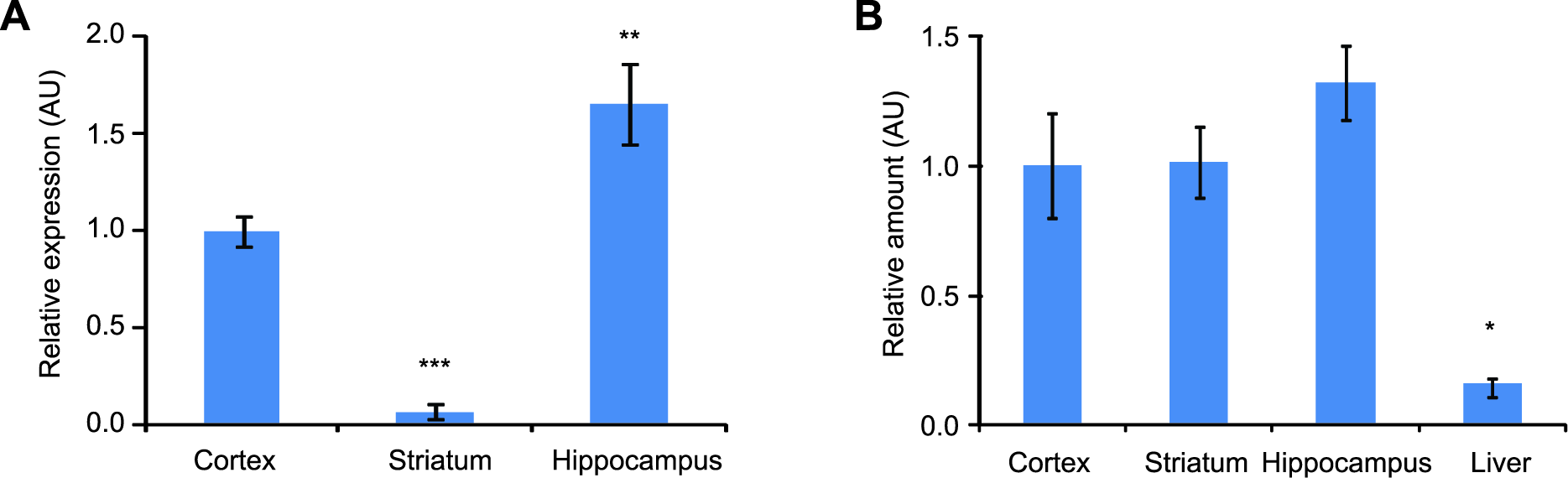

Supplement: Figure S4 — Similar levels of BDNF protein in striatum, cortex and hippocampus despite very low Bdnf mRNA striatal expression. (A) Bdnf mRNA coding region expression in WT mice at 9 weeks between cortex, striatum and hippocampus. Data normalised to Ubc and expressed as fold change of cortex. n≥3/genotype. ** p<0.01 (to cortex) *** p<0.001 (to cortex and to hippocampus) (B) BDNF protein content measured by ELISA in cortex, striatum, hippocampus and liver in WT mice at 9 weeks of age. Data normalised to cortex. * p<0.05; n = 3/genotype. Error bars represent SEM. (TIF) [file pone.0020696.s004.tif]
