# Supplementary material for: Hdac6 Knock-Out Increases Tubulin Acetylation but Does Not Modify Disease Progression in the R6/2 Mouse Model of Huntington's Disease
Source: PLoS One. 2011 Jun 3;6(6):e20696. doi: 10.1371/journal.pone.0020696 (PMC3108987; doi:10.1371/journal.pone.0020696)
Supplement: Table S1 — Statistical analysis of the influence of time (30 min duration) and genotype(s) on the spontaneous motor activity parameters: activity, mobility, rearing and centre rearing presented as p - values calculated via ANOVA General Linear Model with Greenhouse-Geisser correction. (DOC) [file pone.0020696.s005.doc]

|  | **Week** | **Activity** | **Mobility** | **Rearing** | **Centre Rearing** | P = < 0.001 |
| --- | --- | --- | --- | --- | --- | --- |
| **Time** | **5** | <0.001 | <0.001 | <0.001 | <0.001 | P = < 0.01 |
|  | **7** | <0.001 | <0.001 | 0.001 | 0.008 | P = < 0.05 |
|  | **9** | <0.001 | <0.001 | <0.001 | 0.010 |  |
|  | **11** | <0.001 | <0.001 | 0.019 | 0.038 |  |
|  | **13** | <0.001 | <0.001 | 0.045 | 0.054 |  |
| **R6/2 Genotype** | **5** | 0.254 | 0.064 | 0.241 | 0.428 |  |
|  | **7** | <0.001 | <0.001 | 0.527 | 0.338 |  |
|  | **9** | <0.001 | <0.001 | <0.001 | <0.001 |  |
|  | **11** | <0.001 | <0.001 | <0.001 | <0.001 |  |
|  | **13** | <0.001 | <0.001 | <0.001 | <0.001 |  |
| **Time*R6/2 Genotype** | **5** | 0.011 | 0.078 | 0.016 | 0.091 |  |
|  | **7** | <0.001 | <0.001 | <0.001 | 0.002 |  |
|  | **9** | <0.001 | <0.001 | 0.001 | 0.071 |  |
|  | **11** | <0.001 | <0.001 | 0.001 | 0.138 |  |
|  | **13** | 0.009 | <0.001 | 0.063 | 0.171 |  |
| ***Hdac6KO* Genotype** | **5** | 0.771 | 0.946 | 0.421 | 0.380 |  |
|  | **7** | 0.204 | 0.411 | 0.297 | 0.452 |  |
|  | **9** | 0.511 | 0.906 | 0.447 | 0.563 |  |
|  | **11** | 0.272 | 0.378 | 0.876 | 0.781 |  |
|  | **13** | 0.557 | 0.908 | 0.795 | 0.765 |  |
| **Time**Hdac6KO* Genotype** | **5** | 0.053 | 0.070 | 0.815 | 0.515 |  |
|  | **7** | 0.330 | 0.083 | 0.449 | 0.262 |  |
|  | **9** | 0.401 | 0.371 | 0.225 | 0.229 |  |
|  | **11** | 0.808 | 0.747 | 0.133 | 0.192 |  |
|  | **13** | 0.704 | 0.705 | 0.726 | 0.339 |  |
| **R6/2 Genotype**Hdac6KO* Genotype** | **5** | 0.201 | 0.357 | 0.950 | 0.635 |  |
|  | **7** | 0.718 | 0.701 | 0.490 | 0.891 |  |
|  | **9** | 0.907 | 0.746 | 0.032 | 0.091 |  |
|  | **11** | 0.542 | 0.488 | 0.638 | 0.922 |  |
|  | **13** | 0.587 | 0.610 | 0.443 | 0.499 |  |
| **Time*R6/2**Hdac6KO*** | **5** | 0.160 | 0.481 | 0.184 | 0.315 |  |
|  | **7** | 0.019 | 0.022 | 0.167 | 0.325 |  |
|  | **9** | 0.263 | 0.206 | 0.270 | 0.559 |  |
|  | **11** | 0.304 | 0.495 | 0.822 | 0.739 |  |
|  | **13** | 0.189 | 0.490 | 0.556 | 0.493 |  |
